# Hard Corns

**Published:** 1874-04

**Authors:** 


### HARD CORNS.

It has been recommended to peel the corn off with the nail of the finger or thumb, on drawing off the stockings at night. This is tedious and sometimes painful. An incomparably better plan is first to soak the feet in quite warm water for at least a quarter of an hour. This softens the whole corn, when it is very readily detached. Repeat the process as often as the corn grows out again. If the corn is hard and projecting, and the patient cannot afford to throw away the tight boot or shoe which is the cause of the difficulty, cut a hole in a double thickness of buckskin, bind it on the toe so that the hole shall receive the corn. This protects it from pressure, and in time it will fall off of itself.

